# Health Care Worker Perspectives of HIV Pre-exposure Prophylaxis Service Delivery in Central Uganda

**DOI:** 10.3389/fpubh.2022.658826

**Published:** 2022-04-04

**Authors:** Timothy R. Muwonge, Rogers Nsubuga, Norma C. Ware, Monique A. Wyatt, Emily Pisarski, Brenda Kamusiime, Vicent Kasiita, Grace Kakoola Nalukwago, Charles Brown, Agnes Nakyanzi, Monica Bagaya, Felix Bambia, Timothy Ssebuliba, Elly Katabira, Peter Kyambadde, Jared M. Baeten, Renee Heffron, Connie Celum, Andrew Mujugira, Jessica E. Haberer

**Affiliations:** ^1^Infectious Diseases Institute, Makerere University, Kampala, Uganda; ^2^Harvard Medical School, Boston, MA, United States; ^3^Department of Medicine, College of Health Sciences, Makerere University, Kampala, Uganda; ^4^Most At-Risk Populations Initiative, Kampala, Uganda; ^5^STD/AIDS Control Program, Ministry of Health, Kampala, Uganda; ^6^Departments of Global Health and Epidemiology, University of Washington, Seattle, WA, United States; ^7^Gilead Sciences, Foster City, CA, United States; ^8^Center for Global Health, Massachusetts General Hospital, Boston, MA, United States

**Keywords:** healthcare worker, PrEP training, HIV prevention, serodiscordant couples, Uganda, sub-Saharan Africa

## Abstract

**Background:**

Scale-up of HIV pre-exposure prophylaxis (PrEP) services in Uganda is ongoing. However, health care workers (HCWs) may not be aware of PrEP nor what offering this service entails. We explored the impact of standardized HCW training on the knowledge and perspectives of PrEP service delivery in Uganda.

**Methods:**

We recruited HCWs from facilities that offered HIV-related services in Central Uganda. Using the Uganda Ministry of Health curriculum, we trained HCWs on PrEP services. We collected data about PrEP knowledge, preparedness, and willingness to deliver PrEP to multiple key populations before the training, immediately after the training, and >6 months later (exit). We additionally conducted 15 qualitative interviews after the exit survey. Quantitative data were analyzed by Fisher exact test, while qualitative interview data were analyzed inductively.

**Results:**

We recruited 80 HCWs from 35 facilities in urban (*N* = 24, 30%), peri-urban (*N* = 30, 37%), and rural (*N* = 26, 33%) areas. Most HCWs were nurse counselors (*N* = 52, 65%) or medical/clinical officers (*N* = 15, 18%). Surveys indicated that awareness of PrEP increased after the training and remained high. Knowledge of PrEP (i.e., as an effective, short-term antiretroviral medication to use before HIV exposure for people at high risk) generally increased with training, but significant gaps remained, and knowledge decreased with time. Most HCWs recommended PrEP for female sex workers and HIV serodifferent couples, as well as other key populations. We observed increases in the number of HCW who felt their facility was prepared to cater for HIV prevention and provide PrEP, but this view was not universal. HCWs believed in PrEP effectiveness and embraced it as an additional HIV prevention method. Concerns included patient adherence and behavioral risk compensation. HCWs noted challenges in PrEP delivery in terms of inadequate clinic preparedness, infrastructure, staff capacity, and poor attitudes toward key populations by untrained health workers. They felt further training was needed to ensure a smooth scale-up of services without stigmatization.

**Conclusions:**

Standardized training improved knowledge, willingness, and preparedness to offer PrEP services among most HCWs in Central Uganda. Ongoing training will be needed to optimize PrEP delivery services and expand delivery to levels needed for population-level impact.

## Introduction

Oral pre-exposure prophylaxis (PrEP) using tenofovir (TDF) and emtricitabine (FTC) co-formulated as a once-daily pill has demonstrated effectiveness against the acquisition of HIV infection (1, 2) and is recommended for HIV-negative persons at high risk of infection (3). Although PrEP is being scaled up globally, uptake in some settings is suboptimal, partly due to gaps in public health messaging and low awareness among healthcare workers (HCWs) (4). In Uganda, PrEP has been rolled out in a phased manner, first among HIV serodifferent couples and later expanded to other key populations that include men who have sex with men (MSM), sex workers, people who inject drugs, fisherfolk, transgender populations, adolescent girls and young women, and truck drivers (5).

Training of healthcare workers (HCWs) is a critical step in the rollout of new HIV prevention interventions including oral PrEP, especially given concerns about limited knowledge in HCWs and associated reluctance to recommend the use of PrEP to potential users (6, 7). For instance, in a study conducted to investigate perceptions and attitudes about prescribing PrEP among 311 HIV specialists in Italy, a negative attitude toward PrEP was significantly associated with lack of familiarity with information on, and prescription of, PrEP (8). In sub-Saharan Africa, a positive attitude and confidence to prescribe PrEP has been seen among some HCWs who have undergone training on HIV prevention service provision; feedback from these trainings is an important tool for the design of materials, content, and the national approach to training (9–11). Additional gaps in HCW training exist that must be filled to improve quality assurance of PrEP service delivery (12, 13).

The Uganda Ministry of Health (MoH) has made standardized training materials available to promote provision of PrEP (14, 15), yet the impact of this training on HCW PrEP knowledge has not been previously evaluated. We used mixed methods to assess HCW knowledge, preparedness, and willingness to deliver PrEP to key populations in central Uganda before, immediately after, and at least 6 months after training on PrEP service delivery.

## Methods

### The Barriers Study

The Barriers Study was a mixed-methods study designed to assess knowledge of PrEP and identify potential barriers and facilitators of PrEP uptake and adherence among potential PrEP users, as well as to determine HCW knowledge of PrEP and perceptions regarding preparedness and willingness to deliver PrEP services in Central Uganda. Findings on potential PrEP users are described elsewhere (16); this paper focuses on the impact of HCW training. All activities associated with HCWs occurred in English (the official language used in provision of medical care in Uganda).

### Recruitment

At the time of the study (2018), Central Uganda had 504 HIV care and treatment health facilities, including government health centers, sexually transmitted infection/HIV clinics offering HIV testing and treatment services, HIV serodifferent couple community-based organizations, and non-governmental organizations. We stratified health centers in roughly equal proportions by urban, peri-urban, and rural locations within the central region of Uganda from which we purposively selected a sample of 35 health centers that were felt to be representative of planned routine PrEP delivery sites, This site selection approach was based on characteristics of the existing PrEP sites (i.e., Health Center III or Health Center IV that were offering HIV prevention care and treatment to more than 100 persons registered in the facility HIV registers). Six government-supported facilities were already providing PrEP as part of a pilot program and were excluded from this study. We contacted the leadership of the selected health centers and asked them to identify up to three HCWs who were providing HIV prevention, care, and treatment services. These HCWs were selected from the cadres of administration, laboratory personnel, pharmacists, midwives, nurse counselors, and medical/clinical officers (N.B., medical officers have completed undergraduate medical training, whereas clinical officers have a diploma in medicine). Written invitations were sent to the HCWs asking them to take part in the study, which entailed a baseline survey just before a two-day training on PrEP service delivery plus surveys immediately after the training and at least 6 months later (exit). From the sample of HCWs that completed exit surveys, we purposively selected HCWs for in-depth qualitative interviews based on cadre and designation at the health facilities.

### HCW Training

In January 2018, we invited all HCWs to attend a two-day training on PrEP service delivery that took place at the Infectious Diseases Institute training hub in Kampala, Uganda. We conducted three trainings, each of which included representation from the three geographical regions (i.e., urban, peri-urban, and rural). HCW training was led by a national trainer and focused on PrEP knowledge and service delivery based on Uganda MoH guidelines and training materials (5, 14, 15). Educational methods included both didactic sessions and role-plays. Training modules included: (1) PrEP basics (e.g., what is PrEP, differentiating PrEP from post-exposure prophylaxis (PEP) and antiretroviral therapy (ART), who needs PrEP, identifying people at substantial risk of HIV infection; (2) PrEP screening and eligibility (e.g., PrEP eligibility criteria, screening for substantial HIV risk and PrEP eligibility, PrEP contraindications, acute HIV infection); (3) Initial and follow-up visit requirements (e.g., visit procedures, national HIV testing guidelines, PrEP counseling), and; (4) PrEP stigma, side effects, and HIV seroconversion (e.g., creatinine elevation, strategies to minimize PrEP stigma, seroconversion management), as previously described (16).

### Quantitative Surveys

Quantitative survey questionnaires focused on: (1) HIV prevention knowledge and what methods are being used at the facility; (2) PrEP knowledge, attitudes, and willingness to prescribe PrEP, and (3) provision, support, and preparedness for PrEP service delivery. Authors TRM and JEH developed the questions for the surveys based on the national PrEP guidelines (5). Items were pretested among research staff who were not part of the study team for clarity and relevance. Pre-specified options were given for each question, but participants could also indicate other responses. Trained research assistants administered the surveys individually with each HCW.

### Qualitative Interviews

At the study exit visit, we conducted in-depth interviews among selected HCWs that explored their experiences and perspectives on: (1) the PrEP training they had received in the study; (2) their attitudes and knowledge of PrEP, including adherence; and (3) PrEP delivery and implementation, including a comparison of PrEP to other HIV prevention measures. The interview guide (see Appendix 1) was pilot tested among the research team. All interviews were conducted in a private space by social scientists (VK or BK, both trained in qualitative methods) and were scheduled at convenient times and venues. All interviews were audio-recorded, with permission, using a digital audio recorder.

### Quantitative Analysis

We carried out an exploratory analysis, with the sample size reflecting available resources. Given the small sample size, we used Fisher's exact test for all comparisons between and among categorical variables. In *post-hoc* analyses, we used univariable multinomial logistic regression to determine which comparisons were driving the significance. In these analyses, we compared each survey type (i.e., pre-training, post-training, or exit) as the exposure variables and the question response as the outcome. Data analyses were performed using Stata 14 (StataCorp, College Station, TX).

### Qualitative Analysis

Each interview was transcribed by the interviewer and reviewed by a second researcher for quality. We analyzed each interview question inductively (17), utilizing Dedoose software (version 8.0.35). We developed a codebook that allowed for exploration of emergent content reflecting the experiences from the HCW's perspectives. The initial five transcripts were coded independently by four coders (BK, VK, GK, and JM) who reviewed inconsistencies until consensus was reached. The final codebook was then applied to all transcripts. Coded data were sorted by codes, re-reviewed, and grouped based on similar content. Concepts were selected to correspond to and contextualize the quantitative findings. Results are presented below, with illustrations from interview excerpts. Saturation of qualitative findings was achieved. Results were not presented to participants for feedback.

### Ethical Considerations

We obtained ethics approval from the National HIV/AIDS Research Committee (ARC 196), the Uganda National Council for Science and Technology (SS 4277), and Partners HealthCare/Massachusetts General Hospital (2017P000482/PHS). All participants provided written informed consent and their responses were confidentially stored; data collected were de-identified.

## Results

### Participant Characteristics

We invited 108 HCWs, of whom 80 were enrolled for training that took place between 15^th^ and 23^rd^ January 2018. The remaining 28 HCWs did not attend the training as planned for unknown reasons. All 80 participants completed the training and the survey before and immediately after the training. Sixty-one completed the later exit survey; we were unable to locate the remaining 19 participants. The exit surveys were completed between June and November 2018. Characteristics of the 19 participants who we were unable to locate for exit surveys are shown in Table 1. Their characteristics were largely similar to those participating in the exit surveys with the exception that relatively more were midwives compared to nurse/counselors.

**Table 1:** Participant characteristics.

	**Pre- and post- training survey**	**Exit surveys**	***p*** **-value**	**Lost to Follow-up**	**Qualitative**
	**(***N*** = 80)**	**(***N*** = 61)**		**(***N*** = 19)**	**(***N*** = 15)**
**Work location**					
Urban	24 (30%)	18 (29%)	0.81	6 (32%)	
Peri-urban	30 (37%)	26 (43%)		4 (21%)	
Rural	26 (33%)	17 (28%)		9 (47%)	
**Workstation**					
District hospital	20 (25%)	12 (20%)	0.93	8 (42%)	
Health center or dispensary	47 (59%)	38 (62%)		9 (47%)	
National referral hospital	4 (5%)	4 (6%)		0 (0%)	
Private not-for-profit organization	6 (7%)	4 (6%)		2 (11%)	
Non-governmental organization	3 (4%)	3 (5%)		0 (0%)	
**Cadre**					
Administration	3 (4%)	2 (3%)	0.65	1 (5%)	0 (0%)
Medical/clinical officer	15 (18%)	10 (16%)		5 (26%)	4 (27%)
Nurse counselors	52 (65%)	44 (72%)		9 (47%)	9 (60%)
Midwife	4 (5%)	0 (0%)		3 (16%)	2 (13%)
Laboratory personnel	3 (4%)	3 (5%)		0 (0%)	0 (0%)
Pharmacist	3 (4%)	2 (3%)		1 (5%)	0 (0%)
**Workstation experience (years)**					
<3 years	31 (39%)	24 (39%)	0.86	8 (42%)	2 (13%)
3–6 years	32 (40%)	22 (36%)		7 (37%)	10 (67%)
>6 years	17 (21%)	15 (25%)		4 (21%)	3 (20%)
**HIV-related work experience (years)**					
<3 years	23 (29%)	12 (20%)	0.31	9 (47%)	
3–6 years	33 (41%)	24 (39%)		5 (26%)	
>6 years	24 (30%)	25 (41%)		5 (26%)	

Participant characteristics are presented in Table 1. HCWs reflected urban (*N* = 24, 30%), peri-urban (*N* = 30, 37%), and rural (*N* = 26, 33%) areas. Most were nurse counselors (*N* = 52, 65%), while 15 (18%) were medical/clinical officers, 4 (5%) were midwives, 3 (4%) were administrators, 3 (4%) were laboratory personnel, and 3 (4%) were pharmacists. Most (*N* = 47, 59%) were from health centers or dispensaries, 20 (25%) were from district hospitals, and 13 (16%) were from other facilities. Fifteen in-depth interviews were conducted among six nurse counselors, four medical/clinical officers, three pharmacists and two laboratory personnel.

### HIV Prevention Knowledge

As shown in Table 2, before training, HCWs reported that the information they gave to clients about HIV prevention was most commonly condom use (*N* = 74, 93%), abstinence (*N* = 41, 51%), post-exposure prophylaxis (*N* = 35, 44%), ART adherence (*N* = 28, 35%), and safe male circumcision and faithfulness (both *N* = 24, 30%). An increasing proportion of HCWs indicated they gave clients information about PrEP at each subsequent survey (*N* = 15, 19% vs. *N* = 20, 25% vs. *N* = 23, 38%; *p* = 0.04), although this information did not reach most clients. Increases were also seen regarding provision of information about other HIV prevention tools (i.e., abstinence, safe male circumcision, sexually transmitted infections, and elimination of mother to child HIV transmission); however, they decreased to some extent by the exit survey. Notably, several topics, including family planning and routine HIV testing, were not commonly discussed with clients.

**Table 2:** HIV prevention knowledge.

	**Pre-training survey**	**Post-training survey**	**Exit survey**	**Fisher's exact**
	**(***N*** = 80)**	**(***N*** = 80)**	**(***N*** = 61)**	***p*** **-value**
HIV testing and counseling information given to clients about HIV prevention*				
Condom use	74 (93%)	73 (91%)	57 (93%)	0.89
Abstinence	41 (51%)	56 (70%)^∧^	30 (49%)	**0.02**
Post-exposure prophylaxis	35 (44%)	49 (61%)	34 (56%)	0.08
Safe male circumcision	24 (30%)	48 (60%)^∧^	25 (41%)	**0.001**
ART uptake and adherence	28 (35%)	40 (50%)	24 (39%)	0.15
STI treatment	14 (17%)	32 (40%)^∧^	14 (23%)	**0.005**
PrEP	15 (19%)	20 (25%)	23 (38%)^∧^	**0.04**
Faithfulness	24 (30%)	17 (21%)	11 (18%)	0.20
Safer sex/ risk reduction	18 (22%)	17 (21%)	11 (18%)	0.80
EMTCT	4 (5%)	28 (35%)^∧^	11 (18%)^∧^	**<0.001**
Family planning	9 (11%)	12 (15%)	4 (6%)	0.29
Routine testing	7 (9%)	5 (6%)	2 (3%)	0.48
Partner HIV testing and disclosure	4 (5%)	2 (2%)	6 (10%)	0.16
Not sharing sharps	4 (5%)	2 (2%)	5 (8%)	0.31

*STI, sexually transmitted infections; EMTCT, elimination of mother to child transmission*.

* *Multiple response questions; boldface indicates statistical significance (p < 0.05)*.

∧ *Indicates that this value is significant in comparison with the pre-training value in a post-hoc analysis of the Fisher's exact test*.

In qualitative interviews, HCWs indicated their knowledge of HIV prevention through descriptions of the information they provided to persons at risk for HIV. When referencing the period before their training, HCWs talked about combination prevention including condoms, ART for prevention (i.e., prevention of secondary transmission), abstinence, treatment of sexually transmitted infections, HIV testing, and post-exposure prophylaxis.

“*I would emphasize condom use, though it has its challenges because some people pull them off in the process. I would also encourage the couple to adhere well to the drugs and also for the couples to support each other like negative partner to encourage the positive partner to adhere well to the drugs because if the positive person is taking the drugs well, the chances of infection are low….” (Nurse counselor: #04)*

“*…abstain if it is possible. Also to test and treat sexually transmitted infections promptly, and we also tell them to have sex with a partner with whom you have tested for HIV. Also, a person who has encountered a problem, for example when she has been raped or when he has had a needle prick, we encourage that person to take the medicine which is swallowed [PEP] to prevent one from acquiring HIV. (Nurse counselor: #08)*

There was, however, a shift in information given about HIV prevention services after the training on PrEP. In addition to the other options, HCWs described including PrEP to the available HIV prevention messaging, particularly for HIV serodifferent couples.

“*Ever since we got the training, at least discordant couples we have been getting, we have been sharing with them. We have been passing on the information although drugs [PrEP] have not been here. Every time clients come to the health facility, we give them updates about PrEP. We keep telling them that there is a drug [PrEP] that can help the negative person to remain negative. Since we know there is PrEP, we encourage them to bring their partners to test and if found negative they can be offered PrEP and remain negative. The positive partner is started on ARVs on that day and then the negative is started on PrEP”. (Medical officer: #10)*

“*We have had scenarios where some male partners come to us asking for the drug and telling us that, ‘I have come to pick the drug. My wife is HIV positive and I wouldn't want to leave her.' So I always tell them to wait a bit (whispers), and it is a challenge because you are giving out information when you are not yet ready”*. *(Nurse counselor: #05)*

HCWs noted the challenge in generating demand in anticipation of providing PrEP when it was not yet available; however, they were enthusiastic about ultimately adding PrEP to the available HIV prevention tools and expressed a desire to make it available to their clientele.

### PrEP Knowledge and Willingness to Prescribe PrEP

Table 3 indicates knowledge and beliefs about PrEP. We saw a significant and largely sustained increase across the surveys in the knowledge of PrEP as an HIV prevention tool, although nearly half of HCWs did not describe PrEP in this way in the exit survey (*N* = 25, 31% vs. *N* = 48, 60% vs. *N* = 33, 54%; *p* < 0.001). HCWs most commonly saw PrEP as benefitting sex workers and HIV serodifferent couples. After the training, more HCWs also reported PrEP as beneficial for other key populations, such as MSM, fisher folk, and truckers. However, some decreases were seen with time and less than half of HCWs felt these other populations would benefit from PrEP in the exit survey. General knowledge of PrEP was high and increased over time. Most HCWs knew that PrEP should be used during periods of risk for HIV acquisition (*N* = 34, 43% vs. *N* = 73, 91% vs. *N* = 42, 69%; *p* < 0.001). Significant increases were seen in the proportion of HCWs indicating that PrEP was for HIV-negative people (*N* = 31, 39% vs. *N* = 55, 69% vs. *N* = 39, 64%; *p* < 0.001) and that PrEP was to be used before HIV exposure (*N* = 23, 29% vs. *N* = 41, 51% vs. *N* = 22, 36%; *p* = 0.01), although large knowledge gaps in these areas remained. A significant decrease was seen in HCWs who thought PrEP would cause drug resistance (*N* = 38, 61% vs. *N* = 9, 11% vs. *N* = 18, 30%; *p* < 0.001). HCWs felt counseling would be the best way to help clients adhere to PrEP (*N* = 52, 65% vs. *N* = 67, 84% vs. *N* = 47, 77%; *p* = 0.02).

**Table 3:** PrEP knowledge and willingness to prescribe PrEP.

	**Pre-training**	**Post-training**	**Exit survey**	**Fishers exact**
	**survey**	**survey**		***p*** **-value**
	***N*** **= 80**	***N*** **= 80**	***N*** **= 61**	
**HCW ever heard of PrEP** (yes/no)^∧∧^	62 (78%)	80 (100%)	61 (100%)	**<0.001**
**PrEP is a tool for HIV prevention** (yes/no)	25 (31%)	48 (60%)	33 (54%)	**0.001**
HCW knowledge about who can benefit from PrEP* ª	**N = 62**	**N = 80**	**N = 61**	
Discordant couples	48 (77%)	71 (89%)^∧^	53 (87%)^∧^	**<0.001**
MSM	14 (23%)	44 (55%)^∧^	28 (46%)^∧^	**<0.001**
Fisher folk	22 (35%)	38 (48%)^∧^	21 (34%)	**0.03**
Sex workers	51 (82%)	78 (98%)^∧^	56 (92%)^∧^	**0.006**
Transgender individuals	1 (2%)	7 (9%)	0 (0%)	**0.01**
People who use drugs	4 (6%)	14 (18%)^∧^	3 (5%)	**0.02**
Truckers	16 (26%)	41 (51%)^∧^	23 (38%)^∧^	**<0.001**
Sex worker clients	5 (8%)	3 (4%)	3 (5%)	0.93
Sexual violence survivors	1 (2%)	0 (0%)	1 (2%)	0.73
Information about PrEP that HCW give clients*	***N*** **= 80**	***N*** **= 80**	***N*** **= 61**	
PrEP is for HIV-uninfected people	31 (39%)	55 (69%)^∧^	39 (64%)^∧^	**<0.001**
PrEP is before exposure	23 (29%)	41 (51%)^∧^	22 (36%)	**0.01**
PrEP uses ARVs	16 (20%)	17 (21%)	7 (11%)	0.30
PrEP is for people at high risk	9 (11%)	16 (20%)	22 (36%)^∧^	**0.002**
PrEP is short-term	8 (10%)	12 (15%)	7 (11%)	0.61
Effective for HIV prevention	4 (5%)	**14 (18%)**	6 (10%)	**0.035**
PrEP side effects	2 (3%)	2 (3%)	1 (2%)	1.00
PrEP does not prevent STI^∧∧^	0 (0%)	6 (8%)	0 (0%)	**0.004**
**HCW knowledge on duration of PrEP use**	***N*** **= 80**	***N*** **= 80**	***N*** **= 61**	
During periods of HIV risk (yes/no)	34 (43%)	73 (91%)^∧^	42 (69%)	**<0.001**
Life-long (yes/no)	8 (10%)	1 (1%)	2 (3%)	**0.03**
Within 3 months (1-3 months) (yes/no)	7 (9%)	1 (1%)	2 (3%)	0.07
Within 1 year (6 months - 1 year) (yes/no)	2 (3%)	4 (5%)	12 (20%)	**0.001**
Factors not associated with PrEP use* ª	***N*** **= 62**	***N*** **= 80**	***N*** **= 61**	
PrEP can promote unsafe sex	39 (63%)	31 (39%)	32 (52%)	0.23
PrEP causes drug resistance	38 (61%)	9 (11%)^∧^	18 (30%)^∧^	**<0.001**
PrEP has “bad side effects”	35 (56%)	26 (33%)	16 (26%)	0.08
PrEP can immunize one from HIV	11 (18%)	13 (16%)	12 (20%)	0.63
PrEP is taken for 28 days	6 (10%)	8 (10%)	4 (7%)	0.82
PrEP is not very effective at protecting against HIV	6 (10%)	5 (6%)	3 (5%)	0.94
PrEP is given after exposure	4 (6%)	5 (6%)	4 (7%)	0.94
How HCWs would help people adhere to PrEP*	***N*** **= 80**	***N*** **= 80**	***N*** **= 61**	
Counseling	52 (65%)	67 (84%)^∧^	47 (77%)	**0.02**
Reminders	17 (21%)	22 (28%)	23 (38%)	0.10
Pillbox	5 (6%)	8 (10%)	2 (3%)	0.31
Explaining PrEP Benefits and Side Effects	3 (4%)	8 (10%)	3 (5%)	0.30
Follow up visits and calls	3 (4%)	7 (9%)	5 (8%)	0.40
Providing health education on PrEP	1 (1%)	11 (14%)^∧^	4 (7%)	**0.007**
Treatment support groups	3 (4%)	8 (10%)	6 (10%)	0.27

* *Multiple response questions, boldface indicates statistical significance (p < 0.05)*.

ª *Data were missing from some surveys due to nonresponse; the total available number of surveys are noted for each question*.

∧ *Indicates that this value is significant in comparison with the pre-training value in a post-hoc analysis of the Fisher's exact test*.

∧∧ *The Fishers exact p-value was statistically significant but data were too sparse to fit a multinomial regression*.

In the qualitative interviews, HCWs expressed their understanding of PrEP through descriptions of what they told clients in anticipation of providing PrEP. They explained, for instance, that PrEP should be taken daily, at the same time, during periods of “high risk.”

“*The negative partner is the one who takes PrEP. And I always tell the couple the duration of taking PrEP which is for as long as the negative partner is still at risk of getting infected. I encourage the couple to adhere well to the drugs by taking the drugs daily since the couple is at high risk of getting infected and must keep the time of taking the drugs.” (Nurse counselor: #04)*

HCWs also identified key populations at substantial risk of HIV as potential candidates for taking PrEP.

“*We tell them [clients] it's a drug given to a negative person to prevent them from acquiring HIV… it is supposed to be given to people at high risk of getting infection, example of key populations like sex workers, men to men, long distance truckers, fisherfolks, boda-boda men [motorcycle taxi riders] and discordant couples and as long as you are at high risk. (Medical officer: #10)*

HCWs explained the function of PrEP, often in reference to antiretroviral therapy and using analogies to convey their understanding. They indicated trust in its effectiveness, especially if potential PrEP users took it as prescribed.

“*I think that if PrEP is taken as prescribed, it is the best… the active drug in PrEP is the same as what is in ARVs [antiretroviral drugs] and if the drug in the antiretroviral treatment can manage to weaken the virus such that it just sleeps, then I am sure that even this PrEP can prevent one from acquiring HIV.” (Nurse counselor: #08)*

“*We'll tell them that PrEP will help them if you are in a situation that can put you at risk of contracting the virus, for example when the man has been tested and he is found positive and you the woman you do not have the virus you can take it. It saves one from contracting the virus especially if his copies are high and not until the virus in the man's body has been weakened so when she is using PrEP the chances of contracting the virus are very low and she might not contract the virus at all”* (*Nurse counselor: #07*)

Some HCW expressed concerns that PrEP might cause behavioral risk compensation and result in promiscuity or more risky sexual behavior because the risk of HIV transmission is reduced by using PrEP.

“*That is my fear because people who are using PrEP know that they will not acquire the virus so my fear is that it makes someone engage into sexual intercourse with a couple of people.” (Laboratory personnel: #06)*

HCWs were also worried about low adherence among the potential PrEP users, which could lead to HIV infection.

“*My other worry is people might take it as a cure and relax in taking the drug so might have sexual intercourse and test positive when tested for HIV/AIDS. So, this means that they must be counseled very well.” (Nurse counselor: #05)*

Overall, the HCWs indicated a high level of understanding about PrEP and were favorable about its benefits for their clients with high HIV risk, although concerns about risk compensation, stigma, and low adherence reflected hesitancy to prescribe it among some HCWs.

### Provision, Support, and Preparedness for PrEP Service Delivery

As shown in Table 4, HCWs were generally confident that they were well staffed to provide counseling and services for HIV prevention, including PrEP, at their respective facilities after the training. Nearly all HCWs would offer or recommend PrEP if it were available. Stigma related to PrEP service delivery to key populations was low and reduced over time (*N* = 13, 16% vs. *N* = 17, 21% and *N* = 3, 5%; *p* = 0.01). Availability of PrEP as a recommended method of HIV prevention increased over time, but remained low (*N* = 6, 8% vs. *N* = 11, 14% vs. *N* = 13, 21%; *p* = 0.07).

**Table 4:** Provision, support, and preparedness for PrEP service delivery.

	**Pre-training**	**Post-training**	**Exit survey**	**Fishers exact**
	**survey**	**survey**		***p*** **-value**
**How well the facility is staffed to provide HIV prevention services** ª	***N*** **= 62**	***N*** **= 80**	***N*** **= 61**	
Very well	28 (45%)	43 (54%)	44 (72%)^∧^	**0.03**
Somewhat	31 (50%)	34 (42%)	17 (28%)^∧^	
Not at all	1 (2%)	2 (3%)	0 (0%)	
Unsure	2 (3%)	1 (1%)	0 (0%)	
**How well the facility is staffed to cater for PrEP delivery** ª	***N*** **= 61**	***N*** **= 80**	***N*** **= 61**	
Very well	30 (49%)	46 (57%)	45 (74%)^∧^	**0.04**
Somewhat	28 (46%)	30 (37%)	16 (26%)^∧^	
Not at all	0 (0%)	2 (3%)	0 (0%)	
Unsure	3 (5%)	2 (3%)	0 (0%)	
**If PrEP were made available, would you offer/recommend PrEP for potential users?** ª	***N*** **= 60**	***N*** **= 77**	***N*** **= 61**	
Yes	57 (95%)	76 (99%)	61 (100%)	0.15
No	3 (5%)	1 (1%)	0 (0%)	
Stigma in offering PrEP services to key populations*	***N*** **= 80**	***N*** **= 80**	***N*** **= 61**	**0.01**
Yes	13 (16%)	17 (21%)^∧^	3 (5%)^∧^	
No	67 (84%)	63 (79%)^∧^	58 (95%)^∧^	
**PrEP is available as a recommended method of HIV prevention**	***N*** **= 80**	***N*** **= 80**	***N*** **= 61**	
Yes	6 (8%)	11 (14%)	13 (21%)^∧^	0.07
No	74 (92%)	69 (86%)	47 (79%)^∧^	

*Boldface indicates statistical significance (p < 0.05)*.

ª *Data were missing from some surveys due to nonresponse; the total available number of surveys are noted for each question*.

* *HCWs were asked if they felt that either workmates, friends, or their families would stigmatize them if they prescribed PrEP*.

∧ *Indicates that this value is significant in comparison with the pre-training value in a post-hoc analysis of the Fisher's exact test*.

In the qualitative interviews, HCWs from different facilities expressed conflicting ideas about their health facilities' readiness to provide PrEP. Key issues were infrastructure and human resources.

“*Currently, there are some staff members who have been transferred to hospitals and therefore staff has reduced at [name of health facility], but they promised us more staff though they have not yet come. Yes, they have transferred some staff to hospitals, which means that the new staff they will bring will have to be trained about PrEP since they are new people at the site. That can be done. But for the case of infrastructure, we have buildings like the laboratory, pharmacy, and counseling rooms”. (Nurse counselor: #04)*

“*Yes. Although the drugs are there in the clinic already, we cannot start them on PrEP… we have to be with the books, and we have to be with everything set. We cannot just say that ‘Anyway, we have some PrEP. Let me give you some'… We are ready to give, but we don't have the resources to do the documentation yet…We need the books. We need the lockers where we are going to store PrEP.” (Nurse counselor: #09)*

To improve site readiness, HCWs preferred for PrEP to be incorporated into the existing HIV prevention structures and programs, starting from the HIV prevention messages offered during HIV testing counseling.

“*…giving a talk about HTS [HIV testing services], I think even PrEP should be talked about as one of the prevention methods so that everyone will be aware that there are drugs. In case one tests negative, they can take and prevent themselves from getting HIV. It (PrEP) should be part of the package given to the people that are HIV-positive and HIV-negative.” (Medical officer: #11)*

“*I would prefer that it is included in the package that we give people about HTS both at community level and at the health facility too so that someone does not learn about PrEP from here only, but also when out there they can know they can access these drugs”. (Nurse counselor: #01)*

Overall, HCWs felt prepared to offer PrEP from a knowledge perspective, but the practical systems for delivery and integration into routine services were not fully ready in some sites.

## Discussion

In this mixed methods study of HCWs from health facilities offering HIV primary care services across Central Uganda, standardized training based on the Uganda MoH PrEP guidelines greatly improved HCW knowledge about PrEP, although some gaps remained. The training also stimulated incorporation of PrEP into the counseling messages given to clients. HCWs were willing to prescribe PrEP to a variety of key populations, although primarily favoring HIV serodifferent couples and sex workers. Notably, adolescent girls, young women, and the general population were not included as key beneficiaries in the Uganda national guidelines at the time of the study. HCWs generally felt prepared to offer PrEP with most facilities being adequately staffed and resourced for PrEP provision.

We previously reported that potential PrEP users primarily received information about HIV prevention from HCWs (16). This reliance on HCWs for information and the need for accurate knowledge to provide high-quality care highlight the importance of ongoing training as PrEP is scaled up in Uganda and elsewhere. Kenya uses the cascade approach, where national and county level “Trainer of Trainers” (ToT) are taught with a standardized curriculum, who, in turn, pass this knowledge onto other health workers and peer educators. To maintain a high-level quality of service delivery, mechanisms like on-site training and mentorship to offer supportive supervision and ongoing monitoring and evaluation have been put in place. (18–20). Similarly, the Uganda MoH is currently scaling up countrywide HCW training for ToTs (5); in all regions, the trained trainers will then offer support to the HCWs at the facilities. From years of experience with ART service delivery across sub-Saharan Africa, the importance of refresher trainings with accountability is clear, particularly as delivery of key messages can wane with time (13). The MoH thus also plans to offer supportive supervision that entails quarterly refresher training and knowledge checks with the help of a checklist to fill some of the remaining gaps as noted in this study. Although not currently planned, additional skill sets, including motivational counseling, to support clients in selecting and utilizing preferred HIV prevention methods would also be beneficial (21).

The paradigm for HIV prevention has been changed by PrEP. PrEP delivery currently based at health facilities requires sufficient knowledge and confidence of HCWs to talk to clients about antiretroviral medications for HIV prevention. They need to explain transient side effects and give clear recommendations for safety in starting and stopping PrEP to optimize effectiveness. The concern for risk compensation among healthcare workers in our study is also notable, despite numerous studies indicating the overall low prevalence of such behaviors (22, 23). Awareness needs to be raised about this literature to prevent such concerns from limiting PrEP provision. Additionally, potential stigmatizing behavior must be addressed both from the community (24), as well as within the healthcare profession (25, 26), although stigma was reported as low among HCWs in our study. Broadening access to PrEP beyond key populations may be helpful in this regard, and many have called for widespread PrEP availability (12). In addition to training HCWs, the MoH should improve awareness of PrEP through social marketing strategies that involve civil society and engage communities in addressing myths and misconceptions and the championing of PrEP. Such efforts will complement healthcare worker training and help combat challenges to PrEP uptake, such as stigma and structural barriers in care access (27).

Although the HCW training program had many successes in conveying knowledge and guidance for PrEP use, the MoH has not yet provided resources to support all aspects of providing PrEP in all public health facilities. The discrepancies in the quantitative and qualitative data on preparedness may reflect the experience of specific cadres of HCWs. That is, some patient-facing aspects (e.g., counseling by nurses) are ready-to-go, while others (e.g., infrastructure as seen by the clinical officer) are not. The logistics supply chain, in particular, can be a challenge (5, 28), and even procurement of PrEP at the national level has been difficult. PrEP availability in Uganda was initially limited to tenofovir/emtricitabine, which was both costly and not otherwise used for ART under government programs in the country. Tenofovir/lamivudine, however, is a much more practical option, given the pharmacologic equivalence of lamivudine and emtricitabine (22) and the widespread availability of low-cost generic formulations in the country for ART. Efforts are needed to balance the demands for this drug between ART and PrEP in a context of limited resources (10, 23); however, the tenofovir/lamivudine formulation may be critical in promoting large-scale rollout of PrEP in public health facilities (29).

This study had several strengths. First, it is among a few reports of HCW knowledge and beliefs about PrEP in sub-Saharan Africa and reflected a wide variety of cadres, health facilities, and geographic locations. Our approach enabled us to follow trends in specific knowledge and beliefs over time, while also allowing for the emergence of new ideas through open-ended interviews. Our study also has limitations. We evaluated a convenience sample of HCWs selected by the leadership of the facilities, which may have introduced sampling bias. Approximately one-fifth of HCW invited for training did not attend and we were unable to ascertain reasons for non-attendance. The relatively small number of HCWs may have also constrained our ability to see significant effects, and missing data and incomplete follow-up at study exit may have biased our findings. Given the lack of a pre-specified sample size in this exploratory study, our findings should be interpreted with caution. Additionally, we did not directly measure the quality of the information provided by healthcare workers to clients. Finally, none of the chosen facilities was providing PrEP at the time of the study. We purposely chose this population to prepare for the rollout of PrEP; however, experiences may differ when PrEP is provided as part of routine service delivery and its use becomes normalized.

## Conclusions

Standardized training improved knowledge, willingness, and preparedness to offer PrEP services among most HCWs in Central Uganda; however, some deficits remained. There is need for sustainable education models that move beyond traditional didactic learning approaches to support PrEP roll-out. Commitment to HCW training is a critical component of successful scale-up of health service delivery, but frequent staff turnover requires ongoing training, support supervision, and monitoring and evaluation to optimize PrEP delivery services and maintain quality assurance.

## Prior Presentation

These data were reported, in part, at the 16^th^ International Conference on HIV Treatment and Prevention Adherence, 7-9th November 2020, (abstract # 29519).

## Data Availability Statement

The raw data supporting the conclusions of this article will be made available by the authors, without undue reservation.

## Ethics Statement

The studies involving human participants were reviewed and approved by (1). National HIV/AIDS Research Committee (ARC 196), (2). Uganda National Council for Science and Technology (SS 4277), and Partners HealthCare/Massachusetts General Hospital (2017P000482/PHS). The patients/participants provided their written informed consent to participate in this study.

## Author Contributions

TM and JH designed the study and wrote the first draft. RN performed the statistical analyses. NW, MW, and EP designed the qualitative component of the study and wrote the interview guides. VK, BK, GN, and JB did qualitative interviews, transcriptions, coding, and qualitative analysis. All authors contributed to data collection, interpretation of the results, and the writing of the manuscript, and all approved the final draft.

## Funding

This study was supported by the U.S. National Institute of Mental Health (grants R01MH098744 and K24MH114732 to JH). The authors designed and executed the study, had full access to the raw data, performed all analyses, wrote the manuscript, and had final responsibility for the decision to submit for publication. The funder had no role in the design, data collection, analysis, interpretation, or writing of the report.

## Conflict of Interest

The authors declare that the research was conducted in the absence of any commercial or financial relationships that could be construed as a potential conflict of interest.

## Publisher's Note

All claims expressed in this article are solely those of the authors and do not necessarily represent those of their affiliated organizations, or those of the publisher, the editors and the reviewers. Any product that may be evaluated in this article, or claim that may be made by its manufacturer, is not guaranteed or endorsed by the publisher.
