# Supplementary material for: Health Care Worker Perspectives of HIV Pre-exposure Prophylaxis Service Delivery in Central Uganda
Source: Front Public Health. 2022 Apr 4;10:658826. doi: 10.3389/fpubh.2022.658826 (PMC9013815; doi:10.3389/fpubh.2022.658826)
Supplement: Supplementary file 2 [file Data_Sheet_2.pdf]

No	Variable_name	Generic Name	Type	Description	Legal Range	Format / Choices / Display width
1	PTID_Req	PTID	int	Participant ID	001-999	Format = nnn
2	Form_year	FormYear	dt	Form completion Year	Jan - Nov 2018	Format = yyyy
3	SimpleChoice	SurveyType	int	Type of survey:	1,2,3	1 = pre-training<br>2 = post-training<br>3 = exit
4	SimpleChoice	Work location	int	Work location	1,2,3	1 = urban<br>2 = peri_urban<br>3 = rural
5	SimpleChoice	Work station	int	What is the type of the facility where the participant works?	1,2,3,4,5	1 = health center or dispensary<br>2 = district hospital<br>3 = national referral hospital<br>4 = private not for profit<br>5 = NGO
6	SimpleChoice	Cadre	int	What is the role of the participant in the unit?	1,2,3,4,5,6	1 = medical/clinical officer<br>2 = nurse/counselor<br>3 = laboratory personnel<br>4 = pharmacist<br>5 = midwife<br>6 = administration
7	SimpleNumber	Workstation experience	int	For how long has the participant been working in the unit? (years)	0.0-20.0	Format = nn
8	SimpleNumber	HIV-related work experience (years)	int	For how long has the participant been working in an HIV related unit? (years)	0.1-20.0	Format = nn

9	SimpleCheck	HIV information given to clients: safer sex/risk reduction	ck	HIV testing, counseling information given to clients: safer sex/risk reduction		0 = 0<br>1 = 1
10	SimpleCheck	HIV information given to clients: STI treatment	ck	HIV testing, counseling information given to clients: safer STI treatment		0 = 0<br>1 = 1
11	SimpleCheck	HIV information given to clients: PrEP	ck	HIV testing, counseling information given to clients: PrEP		0 = 0<br>1 = 1
12	SimpleCheck	HIV information given to clients: ART	ck	HIV testing, counseling information given to clients: ART		0 = 0<br>1 = 1
13	SimpleCheck	HIV information given to clients: condom use	ck	HIV testing, counseling information given to clients: condom use		0 = 0<br>1 = 1
14	SimpleCheck	HIV information given to clients: SMC	ck	HIV testing, counseling information given to clients: SMC		0 = 0<br>1 = 1
15	SimpleCheck	HIV information given to clients: PEP	ck	HIV testing, counseling information given to clients: PEP		0 = 0<br>1 = 1
16	SimpleCheck	HIV information given to clients: abstinence	ck	HIV testing, counseling information given to clients: abstinence		0 = 0<br>1 = 1
17	SimpleCheck	HIV information given to clients: family planning	ck	HIV testing, counseling information given to clients: family planning		0 = 0<br>1 = 1
18	SimpleCheck	HIV information given to clients: routine testing	ck	HIV testing, counseling information given to clients: routine testing		0 = 0<br>1 = 1
19	SimpleCheck	HIV information given to clients: partner testing and disclosure	ck	HIV testing, counseling information given to clients: partner testing and disclosure		0 = 0<br>1 = 1
20	SimpleCheck	HIV information given to clients: Not Sharing Sharps	ck	HIV testing, counseling information given to clients: Not Sharing Sharps		0 = 0<br>1 = 1
21	SimpleCheck	HIV information given to clients: faithfullness	ck	HIV testing, counseling information given to clients: faithfullness		0 = 0<br>1 = 1
22	SimpleCheck	HIV information given to clients: EMTCT	ck	HIV testing, counseling information given to clients: EMTCT		0 = 0<br>1 = 1
23	YesNo	Heard PrEP	int	Have you ever heard of PrEP?	1,2	1 = yes<br>2 = no

24	SimpleCheck	PrEPBenefit_SDC	ck	PrEP beneficaries: serodiscordant couples		0 = 0<br>1 = 1
25	SimpleCheck	PrEPBenefit_MSM	ck	PrEP beneficaries: men who have sex with men		0 = 0<br>1 = 1
26	SimpleCheck	PrEPBenefit_FF	ck	PrEP beneficaries: fisher folk		0 = 0<br>1 = 1
27	SimpleCheck	PrEPBenefit_sex workers	ck	PrEP beneficaries: sex workers		0 = 0<br>1 = 1
28	SimpleCheck	PrEPBenefit_truckers	ck	PrEP beneficaries: truckers		0 = 0<br>1 = 1
29	SimpleCheck	PrEPBenefit_PWUD	ck	PrEP beneficaries: People who use drugs		0 = 0<br>1 = 1
30	SimpleCheck	PrEPBenefit_transgender	ck	PrEP beneficaries: transgender individuals		0 = 0<br>1 = 1
31	SimpleCheck	PrEPBenefit_sex worker clients	ck	PrEP beneficaries: sex worker clients		0 = 0<br>1 = 1
32	SimpleCheck	PrEPBenefit_raped	ck	PrEP beneficaries: Sexual violence survivors		0 = 0<br>1 = 1
33	SimpleCheck	PrEPDuration_dur3months	ck	PrEPDuration: Within 3 months (1-3 months)		0 = 0<br>1 = 1
34	SimpleCheck	PrEPDuration_dur1year	ck	PrEPDuration: Within 1 year (6 months - 1 year)		0 = 0<br>1 = 1
35	SimpleCheck	PrEPDuration_riskperiod	ck	PrEPDuration: During periods of HIV risk		0 = 0<br>1 = 1
36	SimpleCheck	PrEPDuration_lifelong	ck	PrEPDuration: Life-long		0 = 0<br>1 = 1
37	SimpleCheck	PrEPinformation_PrEP is before exposure	ck	Information about PrEP that HCW give clients: PrEP is before exposure		0 = 0<br>1 = 1
38	SimpleCheck	PrEPinformation_PrEP is short term	ck	Information about PrEP that HCW give clients: PrEP is short term		0 = 0<br>1 = 1

39	SimpleCheck	PrEPinformation_PrEP uses ARVs	ck	Information about PrEP that HCW give clients: PrEP uses ARVs		0 = 0<br>1 = 1
40	SimpleCheck	PrEPinformation_PrEP is for HIV uninfected people	ck	Information about PrEP that HCW give clients: PrEP is for HIV uninfected people		0 = 0<br>1 = 1
41	SimpleCheck	PrEPinformation_ PrEP is for people at high risk	ck	Information about PrEP that HCW give clients: PrEP is for people at high risk		0 = 0<br>1 = 1
42	SimpleCheck	PrEPinformation_Effective for HIV prevention	ck	Information about PrEP that HCW give clients: Effective for HIV prevention		0 = 0<br>1 = 1
43	SimpleCheck	PrEPinformation_ PrEP does not prevent STIs	ck	Information about PrEP that HCW give clients: PrEP does not prevent STIs		0 = 0<br>1 = 1
44	SimpleCheck	PrEPinformation_ PrEP side effects	ck	Information about PrEP that HCW give clients: PrEP side effects		0 = 0<br>1 = 1
45	SimpleCheck	PrEPAdherenceHelp_counselling	ck	How would you help people adhere to PrEP:counseling		0 = 0<br>1 = 1
46	SimpleCheck	PrEPAdherenceHelp_reminders	ck	How would you help people adhere to PrEP:reminders		0 = 0<br>1 = 1
47	SimpleCheck	PrEPAdherenceHelp_pillbox	ck	How would you help people adhere to PrEP:pillbox		0 = 0<br>1 = 1
48	SimpleCheck	PrEPAdherenceHelp_PrEPeducation	ck	How would you help people adhere to PrEP:Doing Health Education on PrEP		0 = 0<br>1 = 1
49	SimpleCheck	PrEPAdherenceHelp_benefit and effects	ck	How would you help people adhere to PrEP: Explaining PrEP Benefits and Side Effects		0 = 0<br>1 = 1
50	SimpleCheck	PrEPAdherenceHelp_Follow up visits and calls	ck	How would you help people adhere to PrEP: Follow up visits and calls		0 = 0<br>1 = 1
51	SimpleCheck	PrEPAdherenceHelp_support groups	ck	How would you help people adhere to PrEP: Treatment Support groups		0 = 0<br>1 = 1
52	SimpleCheck	PrEP not associated with_bad side effects	ck	Factors not associated with PrEP: bad side effects		0 = 0<br>1 = 1
53	SimpleCheck	PrEP not associated with_drug resistance	ck	Factors not associated with PrEP: drug resistance		0 = 0<br>1 = 1

54	SimpleCheck	PrEP not associated with_PrEP can promote unsafe sex	ck	Factors not associated with PrEP: PrEP can promote unsafe sex		0 = 0<br>1 = 1
55	SimpleCheck	PrEP not associated with_PrEP is taken for 28 days	ck	Factors not associated with PrEP: PrEP is taken for 28 days		0 = 0<br>1 = 1
56	SimpleCheck	PrEP not associated with_PrEP is given after exposure	ck	Factors not associated with PrEP: PrEP is given after exposure		0 = 0<br>1 = 1
57	SimpleCheck	PrEP not associated with_PrEP not very effective at HIV protecting	ck	Factors not associated with PrEP: PrEP is not very efective at protecting against HIV		0 = 0<br>1 = 1
58	SimpleCheck	PrEP not associated with_PrEP can immunise one from HIV	ck	Factors not associated with PrEP:PrEP can immunise one from HIV		0 = 0<br>1 = 1
60	SimpleChoice	PrEPWillingness	int	In case PrEP was made available, would you be willing to use it to prevent HIV infection?	1,2	1 = yes<br>2 = no
61	SimpleChoice	Stigma1	int	I would be treated badly at work or get sacked	1,2,3,4	1 = strongly agree<br>2 = agree<br>3 = disagree<br>4 = strongly disagree
62	SimpleChoice	Stigma2	int	I would lose friends	1,2,3,4	1 = strongly agree<br>2 = agree<br>3 = disagree<br>4 = strongly disagree
63	SimpleChoice	Stigma3	int	My family would disown or neglect me	1,2,3,4	1 = strongly agree<br>2 = agree<br>3 = disagree<br>4 = strongly disagree
64	SimpleChoice	Stigma4	int	My community would treat me like an outcast	1,2,3,4	1 = strongly agree<br>2 = agree<br>3 = disagree<br>4 = strongly disagree

65	SimpleChoice	Facility readiness for HIV prevention	int	Generally how well the facility is staffed to cater for HIV prevention?	1,2,3,4	1 = very well<br>2 = somewhat well<br>3 = unsure<br>4 = not at all
66	SimpleChoice	Facility readiness for PrEP delivery	int	Generally how well the facility is staffed to cater for PrEP delivery?	1,2,3,4	1 = very well<br>2 = somewhat<br>3 = unsure<br>4 = not at all
67	SimpleChoice	PrEP available and recommended	int	PrEP is available as a recommended method of HIV prevention	1,2	1 = yes<br>2 = no
68	SimpleChoice	PrEP_HIV prevention tool	int	PrEP is available as a recommended method of HIV prevention	1,2	1 = yes<br>2 = no
