# Supplementary material for: Health Care Worker Perspectives of HIV Pre-exposure Prophylaxis Service Delivery in Central Uganda
Source: Front Public Health. 2022 Apr 4;10:658826. doi: 10.3389/fpubh.2022.658826 (PMC9013815; doi:10.3389/fpubh.2022.658826)
Supplement: Supplementary file 3 [file Data_Sheet_3.PDF]

Appendix 1

Qualitative Interview Guide
Health Care Workers
October 17, 2017

Background

Let's get started by you telling me a little about the work you do at your health care facility.
Where do you work? What is your role? How long have you worked here?
What type of HIV prevention services does your facility offer?
What do you do specifically with your clients for HIV prevention?

PrEP Training

Approximately 3 months ago, you attended a training about pre-exposure prophylaxis (PrEP).
Overall, what were your thoughts about the training?
Looking back, what was the most important thing you learned from the training? What are you reasons for saying this?
What did you find least useful? Why?
Was there anything you wished to learn more about?
What advice would you give the organizers about how to make the training more useful for public health facilities like yours in the future?

PrEP Attitudes and Knowledge

This study is looking at people's attitudes about pre-exposure prophylaxis (PrEP) medication to prevent HIV infection. Prior to that training, what were your thoughts about PrEP?

How did the training changeyour attitudes about HIV prevention, and, in particular, PrEP?

Since participating in the training, what have you done differently when discussing HIV prevention with your clients?
Can you give me an example of that?

How did the training change how you approach HIV prevention?
Probe in depth.
What are the reasons someone would need to take PrEP? Why do you say this?
Do you think PrEP should be made available for all HIV-uninfected individuals in Uganda? Which people/ populations should be able to access PrEP?
Why some groups and not others?

Probe in depth about whether the respondent thinks PrEP is for some groups and not others – i.e., serodiscordant couples, female sex workers, MSM, fisher folk.

Do you believe PrEP is effective at preventing HIV in HIV-uninfected individuals? Why or why not?

How does PrEP compare to other HIV prevention methods, in your opinion?

Probe about specific prevention methods: condoms, abstinence, reduction of partners, male circumcision, PEP, ART.

What is your preferred method of HIV prevention for your clients? In what ways?

PrEP Uptake and Adherence

Do you think there is a need/ demand for PrEP in central region? Why or why not?

How could we increase demand for PrEP?

What is your level of interest in offering PrEP to your clients? Why do you say this?

In what ways did this change after the training?

What would make you (more) enthusiastic about PrEP? Is there a 'simpler' word for enthusiastic?

What are some reasons you would wish to be able to offer PrEP to your clients? Why do you say this?

What are some of your concerns about PrEP? Why do you say this?

For you, what do you imagine would be the challenges of taking PrEP for HIV-uninfected individuals? Tell me why this would be difficult.

What would you do to help your clients adhere to PrEP?

Is there anything else you'd like to say about PrEP?

PrEP Delivery and Implementation

Do you think your facility is equipped to deliver PrEP to its clients? Why or why not?

What are the barriers to offering PrEP in public health facilities – i.e., making PrEP more widely available to Ugandans?

Aside from costs, what are other challenges to PrEP delivery? How might these challenges be overcome?

How do you see PrEP as an HIV prevention strategy fitting into the HIV prevention package offered to clients?

How could we best implement new HIV prevention strategies incorporating the use of PrEP into the existing health system?

For respondents who do not support PrEP, ask: what would change your opinion?

Closing

Thinking back on our discussion today, do you have any final thoughts about what would make you interested in offering PrEP for prevention to your clients?

Is there anything else you would like to tell me about HIV prevention?

Thank you for your time.
